# Elective Revision Surgery in Head and Neck Cancer Patients: A Retrospective Analysis at an Oncology Centre in Porto, Portugal

**DOI:** 10.7759/cureus.50253

**Published:** 2023-12-10

**Authors:** André Sousa-Machado, Eurico Monteiro

**Affiliations:** 1 Ear, Nose, and Throat, Centro Hospitalar Universitário do Porto, Porto, PRT; 2 Medical Education and Simulation, Faculdade de Ciências da Saúde, Universidade da Beira Interior, Covilha, PRT; 3 Otolaryngology - Head and Neck Surgery, Instituto Português de Oncologia do Porto, Porto, PRT

**Keywords:** treatment options, survival, risk factors, revision surgery, head and neck cancer

## Abstract

Introduction

Elective revision surgery in head and neck cancer is crucial for refining and optimizing outcomes, ensuring complete tumor removal, and enhancing both functional and aesthetic aspects, ultimately improving the overall quality of life for patients. The aim of this study is to analyze the overall survival (OS) of head and neck cancer patients who underwent elective revision surgery with or without adjuvant treatment between 2019 and 2022, reflecting on the practice between those years in IPO-Porto, Porto, Portugal.

Methods

This study included 58 patients who underwent 116 major neck surgeries. Multidisciplinary head and neck follow-up records were obtained for every patient. Overall survival and disease-specific survival (DSS) curves were calculated using the Kaplan-Meier method, and statistical significance was determined by the Log-Rank test. We did a retrospective study with an anterograde direction, evaluating the treatments that were previously done by the patients.

Results

The sample comprised 51 males (87.9%) and seven females (12.1%), with a mean age of 59.02±11.014 years. Head and neck pathology was mostly in the larynx (36.2%). The type of the first surgery was mainly directed to the primary tumor in 62.1% (n=36). In the other 37.9% (n=22), surgery was directed to the primary tumor in association with neck dissection. The type of the second surgery was mainly revision surgery. We found that T4 tumors (HR (hazard ratio) = 10.219, p = 0.006) and hypopharynx tumors (HR = 5.306, p =0.035) were significantly associated with inferior OS.

Conclusion

In our sample, we found that a T4 and a tumor located in the hypopharynx were significantly associated with inferior OS in elective head and neck oncologic revision surgery. Disease-free survival in patients undergoing revision surgery in our sample is generally poor. In our sample, there was a statistically significant difference in overall survival between the group who underwent surgery for microscopic evidence of persistent tumor (R1) versus clinical and imagiological tumoral persistency. In our sample, the time between the first and second surgeries wasn't linked with an inferior OS. Further studies with larger populations and prospective design, with longer follow-ups can be the road to a better understanding of this issue.

## Introduction

Head and neck cancer (HNC) refers to a group of tumors originating in the mouth, throat, nose, sinuses, salivary glands, and several other anatomical regions of the head and neck. These tumors, typically squamous cell carcinomas, may develop mainly from thin and flat cells that line the internal surfaces of head and neck regions [1]. The incidence of head and neck cancer varies depending on the location and subtype, with oropharyngeal squamous cell carcinoma being the most common subtype. Risk factors for these cancers include tobacco and alcohol abuse, as well as infection by the human papillomavirus (HPV) [1]. In recent years, the incidence of HPV-related head and neck cancer has been increasing, particularly among younger individuals who do not have a history of tobacco or alcohol abuse.

The epidemiology of these tumors is complex, with variations in incidence, prevalence, and mortality rates observed across different regions and populations. Treatment options typically include surgery, radiation therapy, and chemotherapy, either alone or in combination [1]. Early diagnosis and intervention are crucial for improving survival rates and reducing morbidity. A timely review of surgical specimens is crucial in determining the appropriate staging and treatment. American Joint Committee on Cancer (AJCC) guidelines recommend a maximum turnaround time of 14 days for the pathological evaluation of resected specimens [2]. However, delays in surgical review can have significant implications for patient outcomes, particularly in cases where adjuvant therapy is required. Delays in the initiation of adjuvant therapy have been shown to decrease overall survival in these cancer patients.

In the context of oncological surgery, the implementation of stringent perioperative margin control practices is paramount for ensuring optimal outcomes. By incorporating the use of extemporaneous intraoperative examinations, such as frozen section analysis, surgeons can assess the adequacy of surgical margins in real time. These techniques enable the identification of residual tumor cells at the surgical margins, allowing for immediate resection if necessary. Several studies have emphasized the significance of these practices in improving oncological outcomes and reducing the risk of tumor recurrence [3]. The integration of such margin control strategies into surgical protocols demonstrates a commitment to achieving complete tumor resection and highlights the importance of meticulous surgical techniques in oncologic surgical procedures. In addition, delays in radiation delivery increase tumor recurrence rates and decrease overall survival. Therefore, it’s important for healthcare providers to adhere to established guidelines for surgical review and minimize delays in the initiation of adjuvant therapy to optimize patient outcomes [1].

Available data usually focus on the outcome after primary surgery. Stage and treatment type are strong prognostic factors for all sites. Age ≥80 years was associated with poor survival in oral cavity and larynx cancer [4], and complete tumor removal (macroscopic and microscopic) is a crucial factor for overall survival in head and neck cancer [1]. Macroscopic excision involves removing the entire visible tumor and this type of excision aims to prevent the growing tumor and spreading to other body parts. On the other hand, microscopic excision involves removing any remaining cancer cells after macroscopic excision, an essential aspect because even a small number of remaining cancer cells can cause tumors to recur or metastasize, leading to a worse prognosis [5]. Therefore, both macroscopic and microscopic excision are important to achieve the best possible outcome in head and neck oncology. Complete tumor removal is not always possible, especially if the tumor is located near vital structures. In such cases, other treatment options may be considered for disease control and symptomatic relief [5].

The survival rate for head and neck cancer has risen since 2001, but it still remains about 50%, meaning that half of people with these conditions will die within five years, depending on where cancer first develops [6]. Although local recurrence can be common after surgical treatment, there is limited data available that analyze outcomes after revision surgery in head and neck tumors.

The aim of this study is to analyze the overall survival of head and neck cancer patients who underwent elective revision surgery with or without adjuvant treatment between 2019 and 2022, providing a reflection on the practice between those years in the institution. Considering all these, it’s important to evaluate the effectiveness of revision surgery in head and neck cancer, and the impact on treatment outcomes, quality of life, and survival rates. 

## Materials and methods

Place, duration, and design of the study

We performed a single-center retrospective cohort study, including all consecutive patients who underwent elective revision surgery after their first major head and neck oncologic surgical procedures, from October 2016 to December 2020 (51 months), in IPO-Porto - Porto Oncology Center, Porto, Portugal. The research was conducted according to the principles of the Declaration of Helsinki, and informed consent was waived by the institution.

Evaluation

Only surgeries aimed at primary tumor excision and respective ganglionar chains were considered. Patients with second-primary head and neck tumors were excluded.

Variables evaluated

The following data were collected from medical records: gender and age, tumor location, type and complications of the first surgery, time between first and second surgery, type and complications of the second surgery, reason for revision surgery, and clinical stage.

The aim of all surgeries is to remove cancer with adequate margins of normal tissue with minimal morbidity, knowing that clear margins have an impact on local control. Margin requirements differ according to the tumor origin and functional impact was considered.

In this sense, we divided the previously performed surgeries into 1) surgery targeting only the primary tumor, 2) surgery with concomitant head and neck dissection for removal of lymph nodes, and 3) only head and neck dissection for removal of the lymph nodes. Multidisciplinary head and neck follow-up (FU) was maintained for every patient, and if clinical, macroscopic recurrence was verified, patients were proposed for additional treatments. Revision surgery was defined as a surgery performed within 2 months after the first intervention involving the same anatomical region. We evaluated the course of disease in head and neck cancer patients with indications for elective revision surgery in our centre. We also aimed to find possible factors linked with inferior overall survival (OS) in our patients. We also aimed to compare overall survival in patients who underwent surgery for microscopic evidence of persistent tumors versus clinical and imagiological tumoral persistency. We did a retrospective study with an anterograde direction, evaluating the treatments that were previously done by the patients.

Survival outcome

For overall survival (OS), follow-up (FU) was considered as the time between diagnosis and death with disease (event of interest). Patients alive with disease, patients alive without disease, patients that died without disease, and patients lost to follow-up were excluded. During the FU, patients were considered as being alive with and without oncologic disease and dead with local, regional, or distant disease. The cutoff point for statistical analysis was December 2022, encompassing a minimum FU of 24 months. For overall survival (OS), FU was considered as the time between diagnosis and death with or without the disease (event of interest).

Statistical analysis

Categorical variables are presented as frequencies and percentages, and continuous variables as means and standard deviations. Normal distribution was checked using the Shapiro-Wilk test or skewness and kurtosis. Categorical variables were compared with the use of Fisher’s exact test or the chi-square test, as appropriate. Evaluation time between the first and second surgeries according to staging, the reason for surgery, age, and gender was performed with Cox logistic regression. We compared the evaluation time between the first and second surgeries according to the location of the tumor with the Kruskal-Wallis test, taking into account it was found a non-parametric distribution of data. Overall survival and disease-specific survival (DSS) curves were calculated using the Kaplan-Meier method, and statistical significance was determined by the Log-Rank test. All the analyses were performed in the software IBM SPSS Statistics for Windows, Version 28 (IBM Corp., Armonk, NY, USA), and p-values < 0.05 were considered statistically significant. All reported p-values were two-tailed, with a p-value below 0.05 indicating statistical significance. 

## Results

Sample characterization

In the selected period, 116 major neck surgeries were performed in 58 patients (Table 1). The sample was composed of 51 males (87.9%) and seven females (12.1%), with a mean age of 59.02±11.014 years (range 40-84 years) (Table 1). Head and neck pathology was mostly in the larynx (36.2%). The type of the first surgery was mainly directed at the primary tumor in 62.1% (n=36) (Table 1). In the other 37.9% (n=22), surgery was directed at the primary tumor in association with neck dissection. The type of the second surgery was mainly targeting primary tumor in 41.4% (n=24) (Table 1). In the other 37.9% (n=22), surgery targeted the primary tumor and neck dissection. In 12 patients (20.7%) surgery was directed at neck dissection. 13 patients had complications in the first surgery and 15 in the second (Table 1). The most common complication in the first surgery was post-operative bleeding, with a prevalence of 6.9%. In the second surgery, local wound infection was the most common complication in 6.9% (Table 1).

**Table 1:** Categorical variables of the studied sample

Variable	N (%)
Gender	
Male	51 (87.9%)
Female	7 (12.1%)
Site	
Larynx	21 (36.2%)
Oropharynx and Oral Cavity	17 (28.8%)
Hypopharynx	15 (25.9%)
Nasal cavity	5 (8.6%)
Stage of head and neck cancer pathology	
I	11 (19%)
II	19 (32.8%)
III	16 (27.6%)
IV	12 (20.7%)
First surgery	
Tumor resection	36 (62.1%)
Tumor resection with neck dissection	22 (37.9%)
Complications of the first surgery	
Local wound infection	3 (5.2%)
Post-operative bleeding	4 (6.9%)
Post-operative local hematoma	3 (5.2%)
Other	3 (5.2%)
Reason for revision surgery	
Microscopic disease	22 (37.9%)
Macroscopic disease	36 (62.1%)
Second surgery	
Tumor resection	24 (41.4%)
Tumor resection with neck dissection	22 (37.9%)
Only neck dissection	12 (20.7%)
Complications of the second surgery	
Local wound infection	4 (6.9%)
Post-operative bleeding	3 (5.2%)
Local wound fistula	2 (3.4%)
Other	6 (10.3%)
Adjuvant therapy	
None	27 (46.6%)
Radiotherapy	16 (27.6%)
Chemoradiotherapy	15 (25.9%)

The time between surgeries was 1.27±0.534 months (range 0-2 months), with a median follow-up time of 25.39±14.46 months. Evaluation time between the first and second surgeries according to the location of the tumor was similar according to the Kruskal-Wallis test (H(3)=6.352, p=0.096).

Overall survival

Location

**Figure 1:**
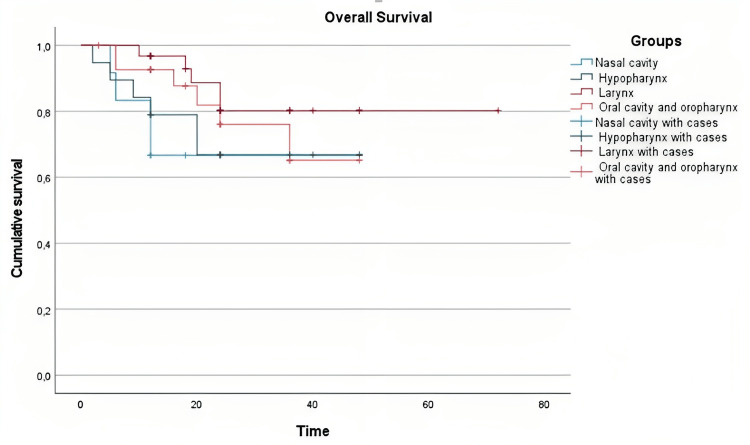
Overall survival in each group evaluated

A total of 58 patients who underwent revision surgery were included in the study, with a median follow-up time of 25.39±14.46 months since revision surgery. Kaplan-Meier curve demonstrated that the probability of survival decreased over time. Log-rank test revealed no statistically significant difference in survival between the groups evaluated, with a chi-square value ranging from 0.053 (p=0.819) to 2.458 (p=0.117) (Figure 1).

Stage

**Figure 2:**
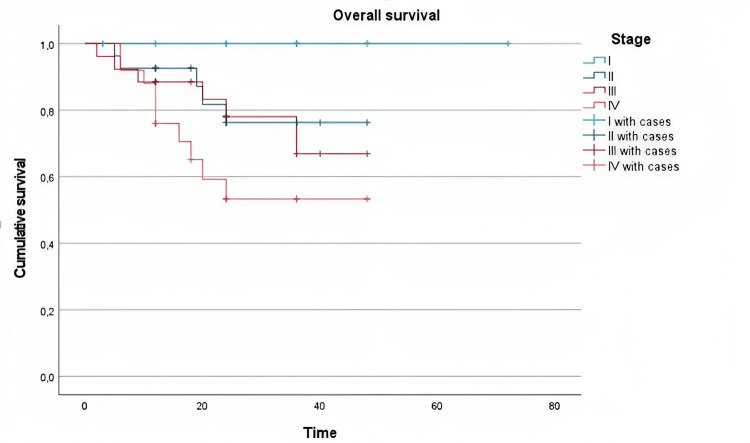
Overall survival in the stages of head and neck cancer pathology evaluated

Kaplan-Meier curve demonstrated that the probability of survival decreased over time. Log-rank test revealed a statistically significant difference in survival between stages I and IV (chi-square=6.307, p=0.012) (Figure 2).

Overall Survival According to Adjuvant Therapy

**Figure 3:**
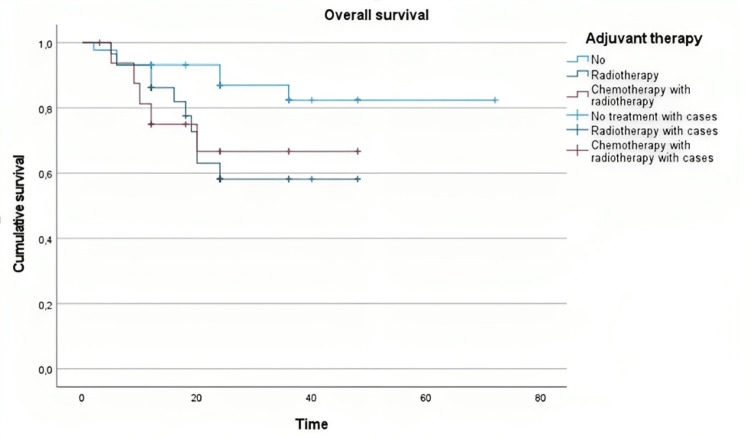
Overall survival according to the adjuvant therapy

Kaplan-Meier curve demonstrated that the probability of survival decreased over time; the Median survival time does not cross the 50% line, and so, the median survival time is not defined. Log-rank test revealed a statistically significant difference in survival between the group who underwent no other treatment and the group who underwent radiotherapy (chi-square=5.341, p=0.021) (Figure 3).

Revision surgery

Macroscopic Versus Microscopic Persistency

**Figure 4:**
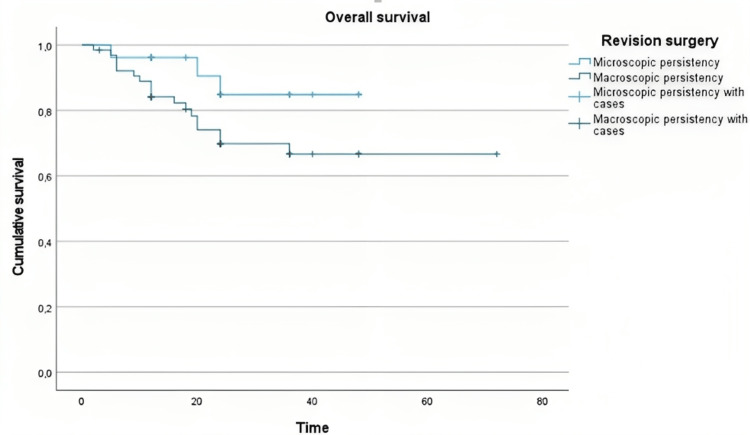
Macroscopic versus microscopic persistency in revision surgery

Log-rank test revealed no statistically significant difference in survival between the group who underwent surgery for microscopic evidence of persistent tumor (R1) versus clinical and imagiological tumoral persistency (chi-square=2.548, p=0.110) (Figure 4).

Cox Logistic Regression Analysis With Predictor Variables

**Figure 5:**
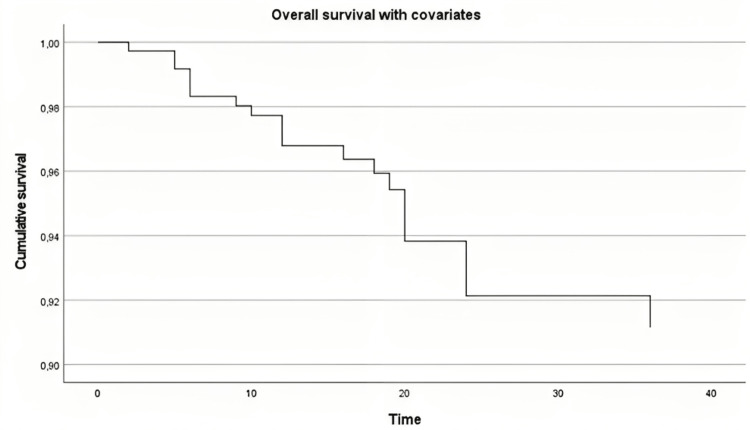
Cox logistic regression analysis to examine the association between OS and four predictor variables OS - overall survival

First, we conducted a Cox logistic regression analysis to examine the association between OS and four predictor variables: reason for revision surgery, age, gender, and tumoral stage. The overall model was not statistically significant (χ2 = 10.739, p = 0.097), indicating that the four preconized predictor variables didn’t contribute to the prediction of the outcome of interest. After adjusting the model for all four predictor variables, we found that the stage (HR = 2.073, p = 0.557), the reason for revision surgery (HR = 1.160, p = 0.281), age (HR = 0.064, p = 0.800), gender (HR = 0.893, p = 0.345) weren’t significantly associated with inferior OS (Figure 5).

**Figure 6:**
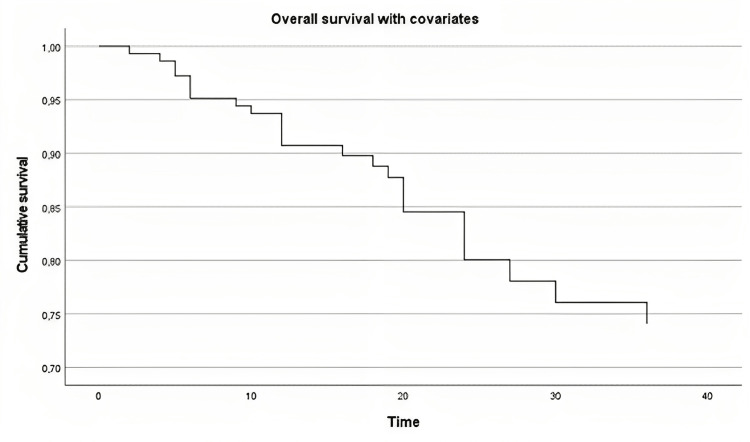
Cox logistic regression analysis to examine the association between OS and the predictor variables: location of the tumor, tumoral stage, and time between first and second surgery, after adjustment of the model. OS - overall survival

Secondly, we conducted a Cox logistic regression analysis to examine the association between OS and the predictor variables: location of the tumor, tumoral stage, and time between first and second surgery. The overall model was not statistically significant (χ2 = 13.079, p = 0.070), indicating that the three preconized predictor variables didn’t contribute to the prediction of the outcome of interest at a significance level of 5%. After adjusting the model for the three predictor variables, we found that a T4 (HR = 10.219, p = 0.006) and a hypopharynx tumor (HR = 5.306, p = 0.035) were significantly associated with inferior OS, while the other predictor variables were not (Figure 6). 

## Discussion

Revision surgery in head and neck cancer refers to subsequent surgical procedures performed in patients who have previously undergone surgery for the treatment of these tumors. This option is often necessary when there is a precocious tumor recurrence following initial surgery [2]. In the present study, the diminished OS time and the non-significant difference in OS between groups are in line with some previous studies in this field [7,8,9]. These results are also in line with previous studies that have reported worse outcomes with increasing disease stage [1,10,11].

The decision to perform revision surgery should be made carefully, taking into account the risks and benefits of the procedures, as well as patient factors such as comorbidities, functional status, and overall prognosis [8]. In patients in which the location and size of the recurrent tumor play a significant role, and if the recurrence is localized and can be surgically removed without causing excessive morbidity, revision surgery can be considered more feasible than other treatments and therefore useful in the management of the patient with head and neck oncologic patient [12].

In addition to surgery, chemotherapy, and radiotherapy are also commonly used in the treatment of head and neck cancer. A clinical trial is currently analyzing the side effects of chemotherapy in patients with locally recurrent head and neck squamous cell carcinoma [13]. This study highlights the poor prognosis associated with this treatment option. However, advances in therapeutics of head and neck cancer such as chemotherapy and radiotherapy may offer some benefits in terms of patient survival. Ongoing research and developments in the field provide hope for improved treatment options and outcomes in the future.

It is worth noting that the impact of head and neck cancer on patients' quality of life is significant [14]. The objective of the study was to evaluate a possible link between the size of the tumors (T), location, and the time between diagnosis and death with disease and the results of elective surgical revision overall survival. According to Cox logistic regression analysis, we found that a higher size of tumor (T4) and hypopharynx localizations were significantly associated with inferior OS after elective surgical revision. This is in line with the literature that states that hypopharyngeal tumors account for 3-5% of cancers with a 5-year survival rate of about 40%. As it often spreads locally, 70-85% of patients are diagnosed at an advanced stage, with this leading to a poor prognosis compared to other head and neck tumor [15-18].

We also aimed to compare overall survival in patients who underwent surgery for microscopic evidence of persistent tumor versus clinical and imagiological tumoral persistency. According to the log-rank test, there was no statistically significant difference in survival between the two groups in a median follow-up time of 25.39±14.46 months. Although there are not many studies that directly compare these two groups, several studies have investigated survival outcomes of revision surgery in head and neck cancer patients. For recurrent HNC (rHNC), only 15-30% of patients are indicative for surgery and the 5-year survival rate is 16-36% [19]. One study reported a 5-year survival rate of 23% for patients who underwent revision surgery for recurrent head and neck cancer in a median time of 28.7 months follow-up, similar to our sample [9]. Another study reported a 5-year overall survival rate of 42.6% for patients who underwent revision surgery for recurrent or persistent head and neck cancer in 132 months of follow-up [10]. This suggests the aggressiveness of the pathology and the limited role that surgery can offer when a late finding is made.

Clinical trials are ongoing to investigate new treatments and improve outcomes for head and neck cancer patients. One is studying the side effects of chemotherapy for patients with locally recurrent head and neck squamous cell carcinoma [11]. Based on our survival analysis, a higher size of tumor (T4) and a hypopharynx localization were significantly associated with inferior OS in elective head and neck oncologic revision surgery. While there are no specific studies that address the relationship between revision surgery and these three predictor variables, there are several studies related to primary hypopharyngeal surgery that are in line with those findings [20,21].

It’s important to note that the results of our study may be limited by its retrospective design and small sample size. Also, analysis based on the histological type was not performed because it would further dilute and fractionate the sample, with less statistical power of the tools used. Further research with larger sample sizes and prospective study designs may be needed to confirm these findings and provide additional insights into the outcomes of revision surgery for head and neck cancer [1-14]. As limitations of our study we consider that the complications rate and evaluation of the quality of life of patients by patient-reported outcome measurements could add more information for this study, although, despite anterograde, its retrospective nature limited the data collection of the variables that weren't considered *ad initium*. The prognostic factors, usually related to this kind of pathology, such as lymph node involvement, metastasis, histologic grade, Human Papillomavirus (HPV) status, smoking, and alcohol consumption, weren't also assessed due to the non-existence of records of all variables in all patients in the database used, although it is known they play a major role in the development of head and neck cancer. Although with a relatively small number of patients, they could play as confounders - and that was the other reason why we opted to consider age, gender, tumour size and location of primary tumour to be evaluated, as they are also prognostic factors recognized by peers.

There is still the need for further research to better understand the predictors of survival in patients who undergo revision surgery for head and neck cancer. Advances in treatment and ongoing research continue to improve outcomes and quality of life for head and neck cancer patients.

## Conclusions

In our sample, we found that a T4 and a tumor located in the hypopharynx were significantly associated with inferior OS in elective head and neck oncologic revision surgery. Disease-free survival in patients undergoing revision surgery in our sample is generally poor. In our sample, a statistically significant difference in overall survival between the group who underwent surgery for microscopic evidence of persistent tumor (R1) versus clinical and imagiological tumoral persistency wasn't verified. In our sample, the time between the first and second surgeries wasn't linked with an inferior OS. Further studies with larger populations and prospective studies with longer follow-ups can be the road to a better understanding of this issue.
